# Detection of Functional Overreaching in Endurance Athletes Using Proteomics

**DOI:** 10.3390/proteomes6030033

**Published:** 2018-09-01

**Authors:** David C. Nieman, Arnoud J. Groen, Artyom Pugachev, Gianmarco Vacca

**Affiliations:** 1 North Carolina Research Campus, Appalachian State University, Kannapolis, NC 28081, USA; 2 ProteiQ Biosciences GmbH, 10967 Berlin, Germany; arnoud@proteiq.com (A.J.G.); artyom@proteiq.com (A.P.); 3 Department of Statistics and Quantitative Methods, Università degli Studi di Milano-Bicocca, 20126 Milan, Italy; g.vacca@campus.unimib.it

**Keywords:** blood proteins, exercise, acute phase response, complement, granulocytes

## Abstract

No reliable biomarkers exist to identify athletes in various training states including functional overreaching (FOR), non-functional overreaching (NFOR), and overtraining syndrome (OTS). Participants (N = 10, age 38.3 ± 3.4 years) served as their own controls and in random, counterbalanced order either ran/cycled 2.5 h (70.0 ± 3.7% VO_2max_) three days in a row (FOR) or sat in the lab (rest) (separated by three weeks; 7:00–9:30 am, overnight fasted state). Participants provided fingerprick samples for dried blood spot samples (DBS) pre- and post-exercise/rest, and then during two recovery days. DBS proteins were measured with nanoLC-MS in data-independent acquisition (DIA) mode, and 593 proteins were identified and quantified. Proteins were considered for the FOR cluster if they were elevated during one of the two recovery days but not more than one of the exercise days (compared to rest). The generalized estimating equation (GEE) was used to identify proteins linked to FOR. A total of 13 proteins was linked to FOR and most were associated with the acute phase response and innate immune system activation. This study used a system-wide proteomics approach to define a targeted panel of blood proteins related to FOR that could form the basis of future NFOR- and OTS-based studies.

## 1. Introduction

Successful training leading to enhanced performance involves cycles of overload and adequate recovery [1,2,3]. A primary goal during training is to avoid the combination of excessive overload and inadequate recovery leading to “overreaching”, defined as a short-term decrement in performance with or without related physiological and psychological symptoms in which restoration of performance takes several days to several weeks [1]. 

“Functional” overreaching occurs when athletes deliberately use a short-term period (e.g., training camp) to increase the training load resulting in short-term performance decrements without serious, long-lasting psychological or other negative symptoms [2]. “Functional overreaching” (FOR or short-term overreaching) will eventually lead to an improvement in performance after recovery. Non-functional overreaching (NFOR or extreme overreaching) occurs when athletes train beyond their ability to recover with concomitant performance decrements and psychological disturbances that include decreased vigor and energy, increased fatigue, and loss of desire to train [1]. NFOR can result in a prolonged recovery time with sleep disturbance, elevated resting heart rate, illness, and psychological stress. A hallmark feature of FOR and NFOR is the inability to sustain intense exercise for a prolonged period of time. NFOR can easily progress to the overtraining syndrome (OTS), and athletes with OTS may take months or possibly years to completely recover.

There is a strong demand for diagnostic tools to identify athletes in various training states for FOR, NFOR, and OTS [1,3]. A reliable biomarker for FOR, NFOR, and OTS should be sensitive to the training load and occur prior to the establishment of OTS. Additionally, changes in the biomarker in response to acute exercise should be distinguishable from chronic changes, and be relatively easy to collect and measure [1]. No established objective biomarkers currently exist for FOR, NFOR, or OTS, and subjective measures are regarded as superior to physiological measures such as plasma hormones and cytokines, energy homeostasis, and exercise workload monitoring [4,5,6,7]. 

Proteins are the main components of the metabolic pathways of cells, and the large-scale measurement of the structure and function of proteins in a tissue or organism is highly useful in the identification of candidate biomarkers for various disease processes and drug treatments [8]. Proteomics, however, has seldom been used in exercise-based, human studies [9], despite the indication from multiple studies with race horses and dogs that serum amyloid A (SAA) and other acute phase response proteins are useful indicators of exercise-induced muscle damage and exhaustion, and poor performance [10,11,12,13,14,15,16,17]. Petibois et al. [18] suggested that acute phase proteins be considered in the biochemical model of the overtraining process based on seminal global metabolic response studies using Fourier-transform infrared spectrometry.

The purpose of this study was to determine if two different clusters of proteins could be identified through global proteomics procedures to define acute compared to chronic physiological changes related to FOR. The goal was to use a simple, practical blood collection measure (fingerprick dried blood spot samples) in developing a targeted proteomics panel of post-FOR chronically expressed proteins that could form the basis for NFOR- and OTS-based studies. Label free targeted proteomics, in this case Data Independent Acquisition (DIA), was utilized in this study because this method generates a record of all detectable fragments of peptides in a sample, combining the advantages of SRM (reproducible) and shotgun analysis (high throughput).

## 2. Materials and Methods

### 2.1. Participants

Study participants included ten healthy, trained, male endurance runners or cyclists, ages 23–50 years. Participants agreed to train normally, stay weight stable, and avoid the use of large-dose vitamin/mineral supplements (above 100% of recommended dietary allowances), herbs, and all medications during the project. All subjects voluntarily signed informed consent forms, and study procedures were submitted to and approved by the Institutional Review Board at Appalachian State University. 

### 2.2. Baseline Testing

Study participants came to the North Carolina Research Campus, Human Performance Lab (Kannapolis, NC, USA) for baseline testing one to two weeks prior to the overreaching segment of the study. Participants reviewed and voluntarily signed the consent form, and supplied training history information. Participants that were runners (N = 3) were tested for VO_2max_ using graded exercise tests on a treadmill, and cyclists (N = 7) on a Lode cycle ergometer (Lode Excaliber Sport, Lode B.V., Groningen, The Netherlands). Continuous metabolic measurements were made with the Cosmed CPET system (Rome, Italy). Body composition was measured using the BodPod system (Life Measurement, Concord, CA, USA). 

### 2.3. Research Design for the Randomized Trials

**Figure 1:**
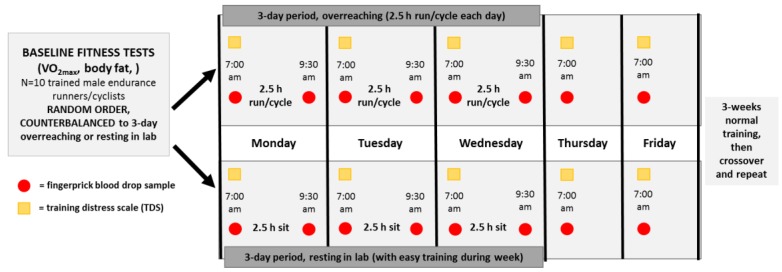
Research design with study participants (N = 10) randomized to 3-day periods of 2.5 h/day running/cycling or sitting and two days resting recovery, with crossover to the counterbalanced condition after a 3-week washout period. Fingerprick blood samples were collected pre- and post-exercise/sitting sessions during each 3-day period, and at 7:00 am the following two mornings (overnight fasted state). The Training Distress Scale (TDS) was administered at 7:00 am each of the five mornings in the lab.

Participants served as their own controls and in random order engaged in a 3-day period of functional overreaching (2.5 h/day, running/cycling) or a 3-day rest period in the Human Performance Lab (Figure 1). Participants reported to the lab in an overnight fasted state, provided a fingerprick sample, and completed the Training Distress Scale (TDS), a 19-item self-reported questionnaire that calculates training distress and performance readiness [19]. At 7:00 am, participants started running/cycling on laboratory treadmills or their own bicycles on CompuTrainer Pro Model 8001 trainers (RacerMate, Seattle, WA, USA) at 70% VO_2max_, or sat in the lab for 2.5 h in an adjoining room to the lab. Heart rate, rating of perceived exertion (RPE), oxygen consumption (Cosmed CPET metabolic system), and ventilation were measured and recorded every 30 min during the 2.5 h exercise bouts. Participants consumed 2–3 mL/kg water every 15 min, and no other food or beverages were consumed during the 2.5-h bouts. An additional fingerprick sample was collected immediately post-exercise. Participants repeated these procedures for two additional days (thus, three 2.5-h exercise bouts or rest periods on Monday, Tuesday, Wednesday), and then returned to the lab to provide fingerprick blood samples and TDS responses on Thursday and Friday mornings at 7:00 am. After a 3-week period, participants crossed over and repeated the counterbalanced procedures. During the 3-day period when participants sat in the lab, moderate but not intensive training regimens were allowed.

### 2.4. Proteomics Procedures

In this study, dried blood spot (DBS) specimens were collected via fingerprick onto standard blood spot cards (Whatman^®^ protein saver cards, Sigma-Aldrich, St. Louis, MO, USA) and dried overnight. Samples were shipped to Biognosys (Schlieren, Switzerland) for global proteomics analysis [20,21]. A puncher was used to remove the middle of the DBS, and proteins were solubilized, reduced and alkylated, and digested into peptides using trypsin. Samples were cleaned up using C18 columns and dried down. To reduce variance, all 16 samples for each athlete (batch) were randomized and subsequently measured consecutively by mass spectrometry (MS). MS sensitivity and precision were monitored using a pooled sample of all DBS samples, with injections before, after, and twice during each batch. MS analyses were performed on a Q-Exactive mass spectrometer (Thermo Fisher Scientific, Waltham, MA, USA) coupled to a nanoLC autosampler. 1 µg of DBS peptide of each sample was injected and peptides were separated with reverse phase nanoLC chromatography. All samples were measured with data independent acquisition mode (DIA).

### 2.5. Data Processing

The DIA files were processed using Spectronaut™ software (Biognosys). Spectronaut was also used to calculate the false discovery rate (FDR) of identified peptides and a cut-off of 0.01 was taken across all samples. For each protein, the three most abundant peptides were used for quantitative analysis (in case more than three peptides per protein were identified).

### 2.6. Statistics

The statistical methodology used to detect protein responses to exercise involved the use of Generalized Estimating Equations (GEE) and Generalized Linear Mixed Models (GLMM) at single protein levels. The interaction between time of the measurement and condition status (exercise, rest) was used as a categorical predictor. In the current study, GEE was the preferred choice given the research design (2-arms, randomized, crossover) and the sample of ten athletes. For the statistical power simulation test, GLMM was used. In this study, the data were first corrected for potential batch effects, prior to normalization relative to the maximum value in each row. To account for technical variability, the significance threshold (*p*-value) was set to 0.01 if the coefficient of variation (CV) in the z-score of a given protein in technical repeat samples exceeded 15% across athletes and time points. Otherwise the *p*-value significance level was set at *p* ≤ 0.05. The simulation generated artificial, normally distributed data using the technical variance of the original data. Different levels were derived from twelve proteins randomly selected from the subset of athletes (from 10 to 50 with steps of 10), with the assumption that the effect would be notable in 80% of the athletes at each time point. Bonferroni correction was applied assuming a final panel of five proteins. After estimating the GEE and GLMM models, pairwise comparisons between each time-by-condition level were calculated, and the Tukey correction for multiple comparison was applied to adjust the significance level. The GEE analysis of the original data and the GLMM analysis of simulated data delivered identical results for given proteins.

### 2.7. Protein–Protein Interaction Network Analysis

Proteins expressed acutely following each of the three 2.5-h exercise sessions, and those expressed on day 1 and/or day 2 of recovery were mapped onto STRING v10 to build two protein–protein interaction networks. STRING v10 (search tool for the retrieval of interacting genes/proteins) is a database of known and predicted physical/functional protein associations based on genomic context, high-through put experiments, co-expression and previous knowledge (http://string-db.org/) [22]. 

## 3. Results

Study participant data (N = 10 males) are summarized in Table 1. Metabolic monitoring of the three 2.5-h exercise sessions showed that the athletes averaged a heart rate of 140 ± 4.3 beats/min (79.6 ± 2.0% maximal heart rate or HR_max_), oxygen consumption of 29.4 ± 0.9 mL kg^−1^ min^−1^ (70.0 ± 1.2% maximal oxygen consumption or VO_2max_), and ventilation of 65.1 ± 1.9 L/min. The rating of perceived exertion (RPE) averaged 15.4 ± 0.3 units at the end of each session (rating of “hard”). No significant differences were found between the runners (N = 3) and cyclists (N = 7) for the data listed in Table 1 and exercise performance data, and all analyses were conducted for the combined group of these 10 athletes. 

The simulation using GLMM modelling with 34 proteins showed that a relatively low number of participants was needed to show significant changes in protein up and down regulation, supporting the use of 10 athletes in this randomized, crossover trial. Data consistency was monitored through several pooled samples, and the results supported a reproducible protein quantitation. 

**Figure 2:**
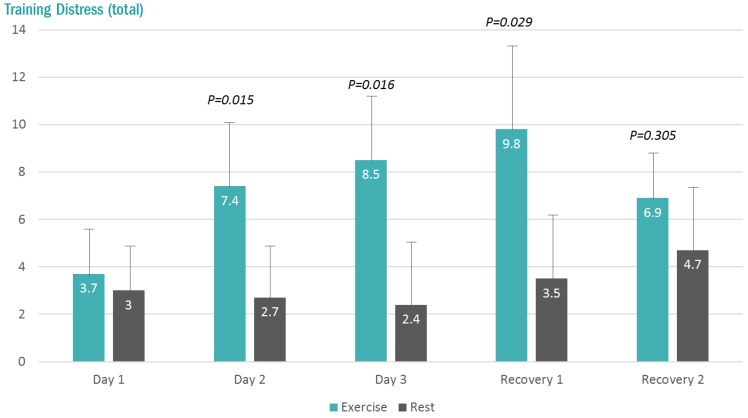
Changes in the total Training Distress Scale (TDS) scores with exercise and rest conditions (interaction effect, *p* < 0.001). *p*-values, change pre-to-post change exercise compared to rest day.

The total Training Distress Scores (TDS) were higher at 7:00 am (pre-lab sessions) in the exercise compared to rest trials on the second and third days, and the first day of recovery (interaction effect, *p* < 0.001) (Figure 2).

**Figure 3:**
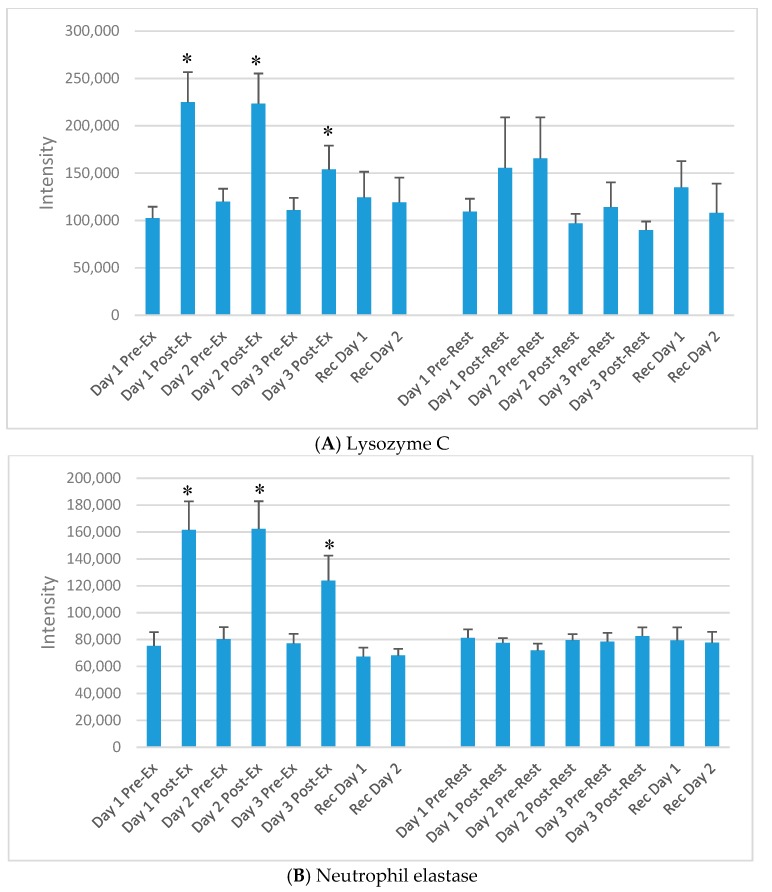

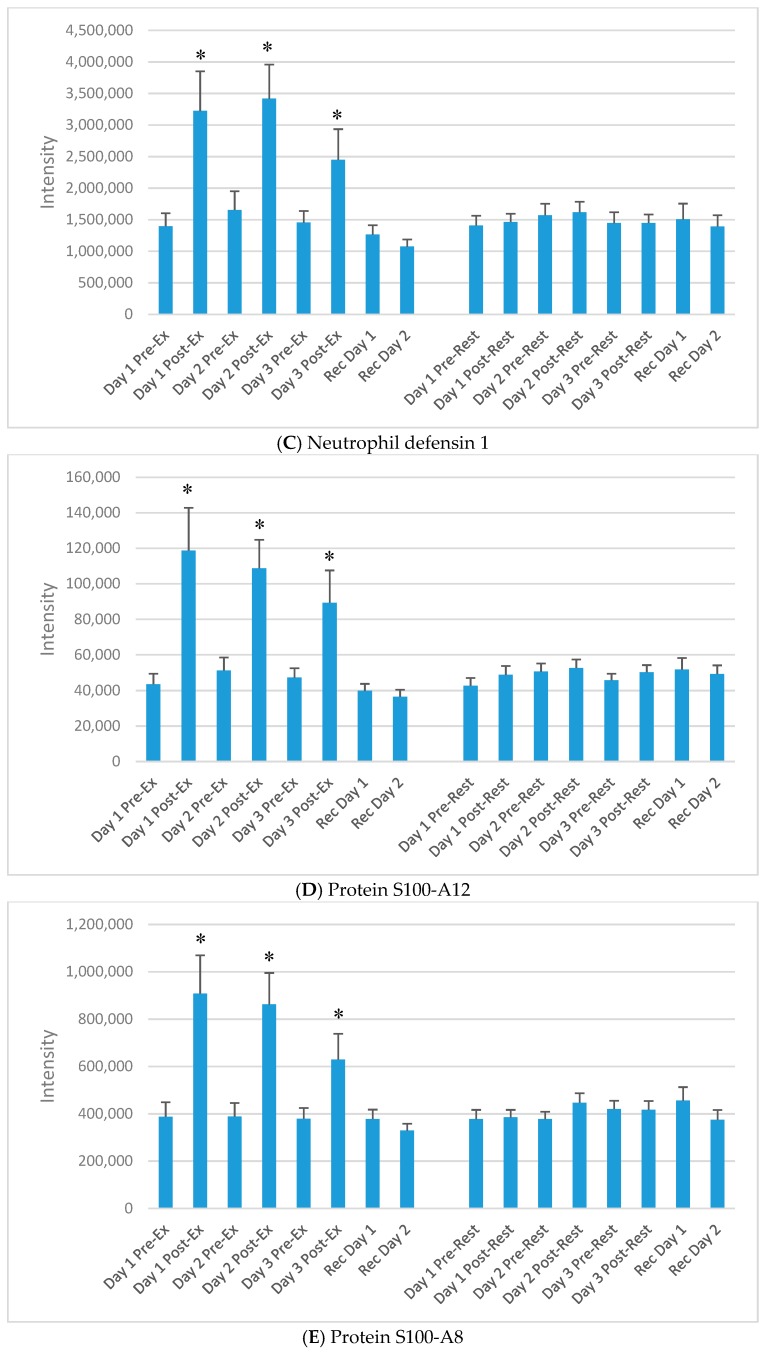

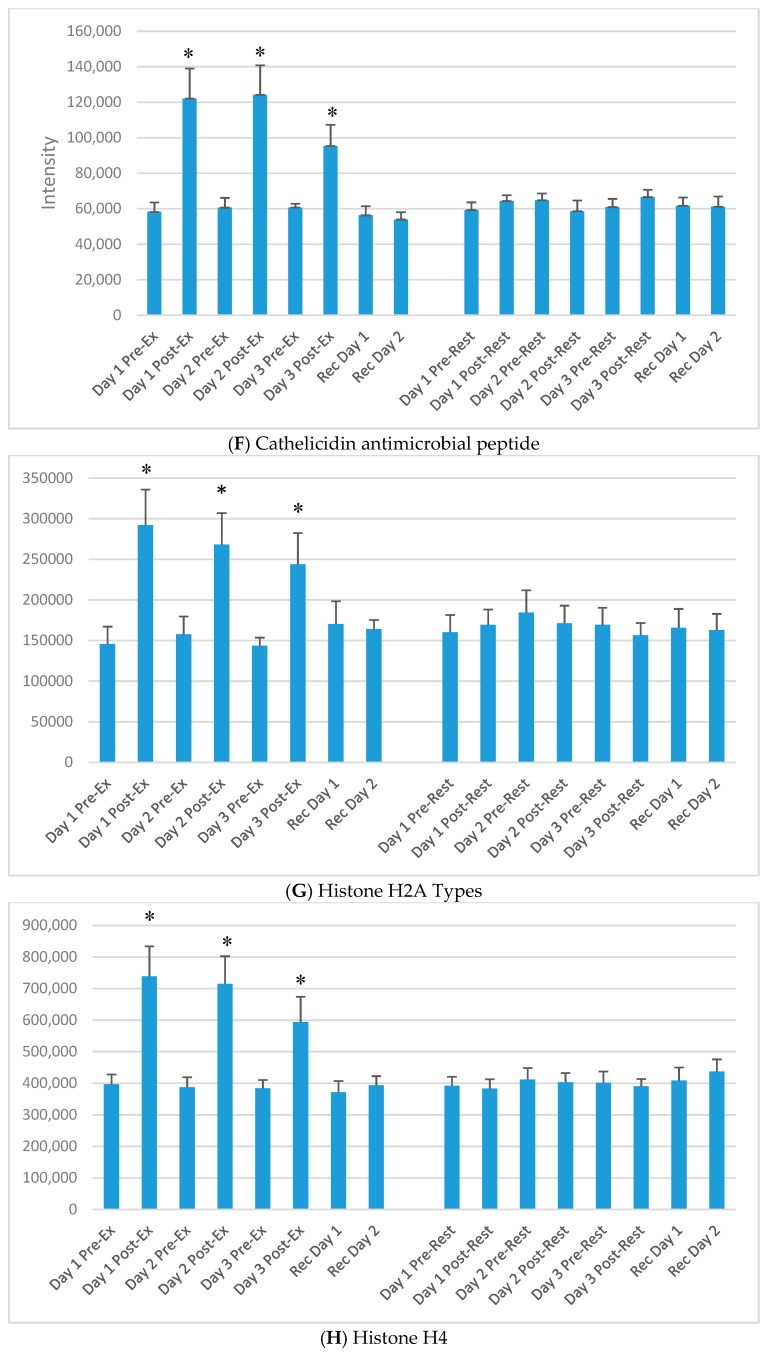
Selected plasma proteins (from Table 2) increasing acutely each day of the 3-day exercise period compared to rest. (**A**) Lysozyme C; (**B**) Neutrophil elastase; (**C**) Neutrophil defensin 1; (**D**) Protein S100-A12; (**E**) Protein S100-A8; (**F**) Cathelicidin antimicrobial peptide; (**G**) Histone H2A types; (**H**) Histone H4. * *p* < 0.05, change pre-to-post change exercise compared to rest day.

The DIA approach using the dried blood spot samples (16 samples per athlete) resulted in the identification of 6499 precursors. Protein inferences (discarding the non-unique peptide hits and assigning the peptides to the proteins of origin) resulted in a total number of 593 proteins (intra-batch technical median CV of 22%, batch correction using the R package Combat) (see Table S1) [20,21]. Of the 593 identified proteins, 60 increased significantly immediately post-exercise on day 1 (of the 3-day exercise period) compared to the rest condition. Of these, 30 were related to immune function. Table 2 lists 15 of the proteins that increased significantly immediately post-exercise after each of the three days of exercise compared to rest, and most were related to immune function. The median technical CV of the listed proteins was 6%, with a range of 3–8%, except for O95810 with 23%. Figure 3A–H) compares exercise and rest intensity data for eight of these 15 immune-related proteins.

**Figure 4:**
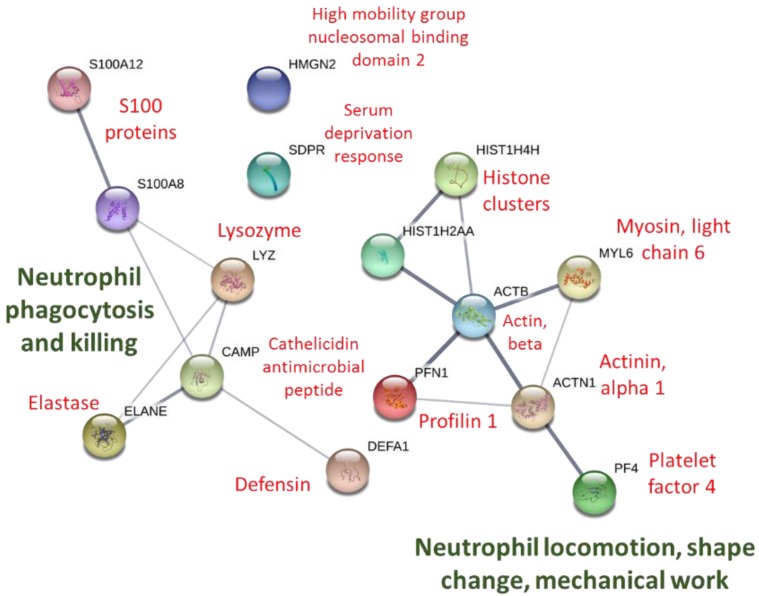
STRING protein–protein interaction graph using immune-related proteins listed in Table 2. The thickness of the network lines indicates the strength of data support (https://string-db.org).

STRING protein–protein interactions using proteins listed in Table 2 are depicted in Figure 4. Biological process pathways identified in STRINGS for the 15 proteins included four gene sets involved with killing of cells of other organisms, four to six with the defense response to bacterium, fungus, and other organisms, six to seven with chemotaxis and locomotion, and seven to eight with the immune system response (all FDR < 0.015). 

**Figure 5:**
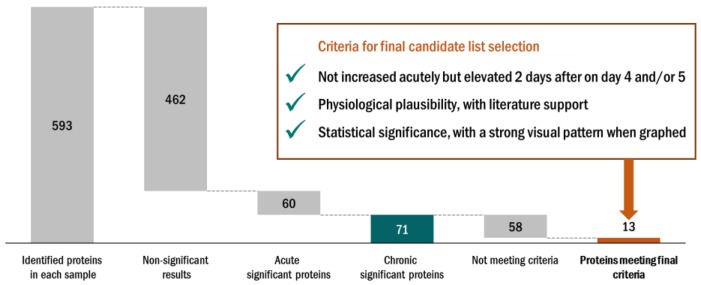
Selection process to determine the protein cluster (N = 13) associated with functional overreaching (FOR).

**Figure 6:**
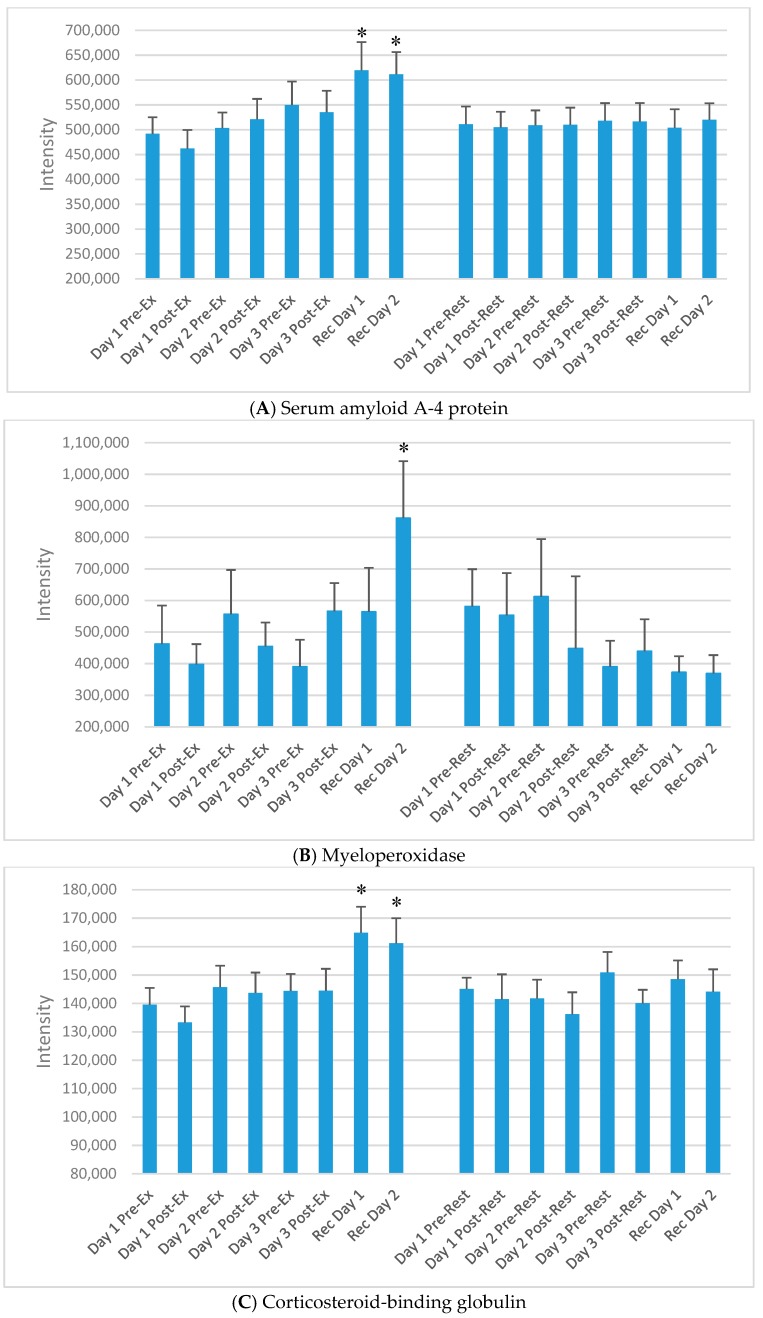

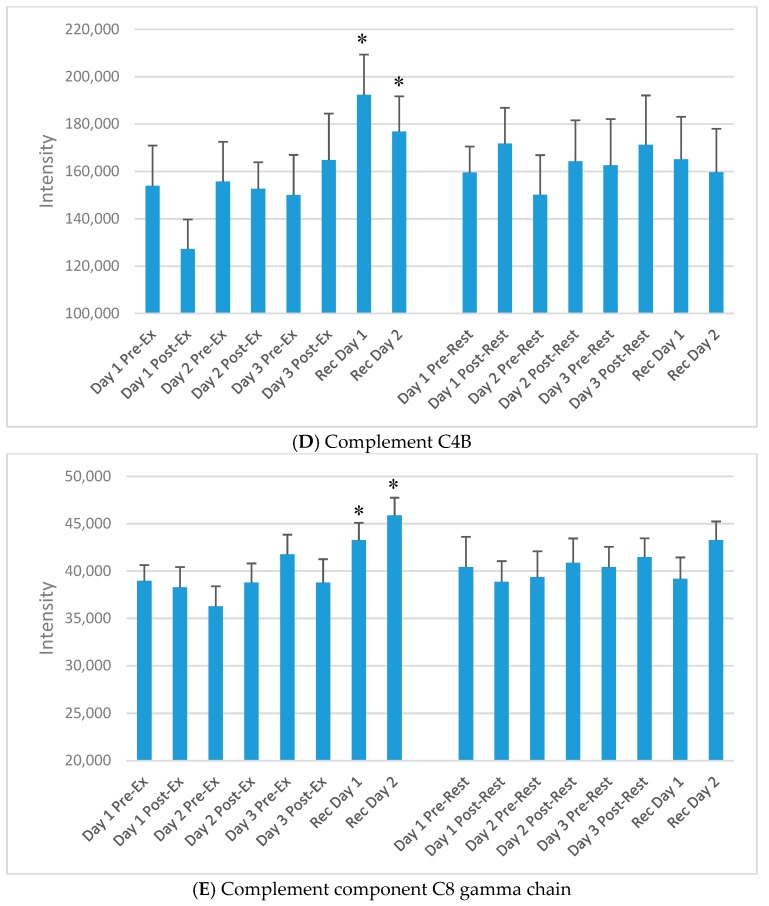
Selected plasma proteins increasing during day 1 and/or day 2 of recovery from the 3-day exercise period compared to rest, but not acutely immediately post-exercise. (**A**) Serum amyloid A-4 protein; (**B**) Myeloperoxidase; (**C**) Corticosteroid-binding globulin; (**D**) Complement C4B; (**E**) Complement component C8 gamma chain. * *p* < 0.05, change pre-to-post change exercise compared to rest day.

“Chronic proteins” in this study were defined as proteins that did not increase or decrease acutely after the three exercise versus rest bouts, but increased only on the morning of day 1 and/or day 2 of recovery. Of the 593 proteins, 71 chronic proteins were identified through GEE modelling. Three other criteria were applied to narrow down this list of proteins to those most strongly associated with the recovery period from the FOR exercise period. An overview of this approach is described in Figure 5, with emphasis on recovery day expression, those that showed a clear exercise versus rest pattern when graphically displayed, and proteins that had literature support and biological plausibility. After application of these criteria, 13 chronic proteins were included as listed in Table 3. Of these, at least 11 were related to immune function. Intensity data for five of the 13 proteins from Table 3 are presented in Figure 6A–E.

**Figure 7:**
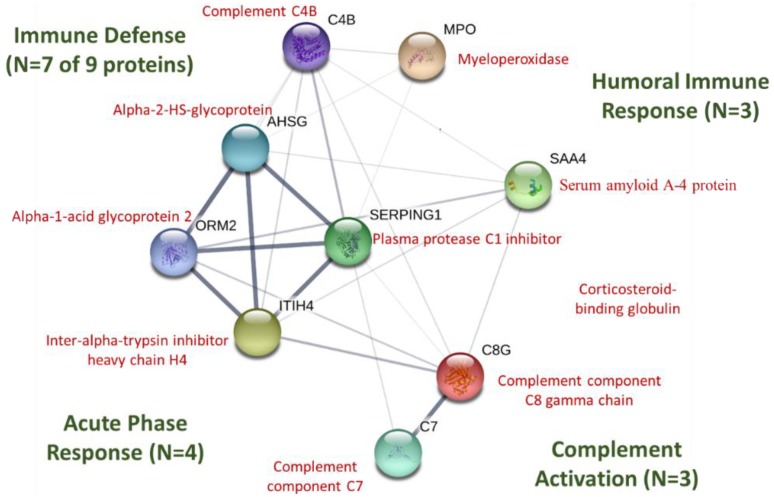
STRING protein–protein interaction graph using immune-related proteins listed in Table 3. The thickness of the network lines indicates the strength of data support (https://string-db.org). P01834 (Ig kappa chain C region) and P04220 (Ig mu heavy chain disease protein) for humans were not listed in STRING.

STRING protein–protein interactions using the proteins listed in Table 3 are depicted in Figure 7 (https://string-db.org). P01834 (Ig kappa chain C region) and P04220 (Ig mu heavy chain disease protein) for humans were not listed in STRING. Of the nine proteins networked in Figure 5 (all FDR < 0.001), seven were included in the gene set for the immune defense response (biological process), four in the acute phase response, three in complement activation, and three in the humoral immune response mediated by circulating immunoglobulins.

## 4. Discussion

Using a randomized, crossover design, ten male runners and cyclists sat in the lab or exercised intensely for 2.5 h each morning in an overnight fasted state for three days in a row. Total distress scores showed that the 3-day exercise period increased psychological distress to levels expected during functional overreaching [19]. Dried blood spot (DBS) samples were collected pre- and post-exercise/rest, and at 7:00 am the following two mornings, and the use of this practical blood collection method allowed the acquisition of a large number of samples with minimal discomfort to the athletes. DBS samples offer many advantages, especially in an athletic setting, as a sample format including ease and safety of transport and handling [23]. Analytes preserved within the DBS are stable for long time periods at ambient conditions and can be eluted in solvents for later proteomics analysis. 

Global proteomics procedures from the DBS samples showed that of 593 proteins identified, 60 proteins increased significantly after the 2.5 h exercise bout on day 1 (of the 3-day exercise period) and 15 after each of the three days of exercise compared to rest. STRING protein–protein interactions showed that these 15 proteins expressed acutely post-exercise were involved with an activated immune system response including pathogen defense and immune cell chemotaxis and locomotion. Some of these proteins involved in the acute response to exercise have been identified previously, especially neutrophil elastase and protein S100-A8 [16,17,24,25]. S100-A8/A9 (calprotectin) is released primarily from activated neutrophils, is found in high levels in human blood samples (~5 mg/L), promotes phagocyte migration and functions as an alarmin and endogenous danger-associated molecular pattern (DAMP), and is regarded as a cardiovascular disease (CVD) risk factor when systemically expressed [26]. S100-A12 is a ligand for receptor advanced glycation end products (RAGE), promotes phagocyte chemotaxis, and is a potent stimulator of acute inflammation [27,28]. 

Polymorphonuclear neutrophils are the first cells recruited to inflammatory sites following exercise [28], and neutrophil elastase is one of three serine proteases stored in granules that act in combination with reactive oxygen species to help degrade engulfed microorganisms and debris [29]. Neutrophils read chemotactic peptides released from damaged cells or bacteria, and then respond by increasing the nucleation and polymerization of actin filaments. Profilins are small (12 to 15 kDa) and abundant proteins that have been found in all eukaryotic cells tested [30] and are involved in the dynamic turnover and restructuring of the actin cytoskeleton. Thus, post-exercise increases in profilin-1 and actin (cytoplasmic 1) appear to represent the increase in neutrophil actin filament polymerization that supports migration to involved tissues [31]. 

The primary purpose of this investigation was to identify a targeted panel of post-FOR chronically expressed proteins that could be utilized and validated in future overtraining-based investigations. Our analysis found that 13 of the 593 identified proteins did not increase acutely post-exercise, but increased on the morning of day1 and/or day 2 of recovery. STRING protein–protein interactions showed that most of these proteins were involved in the immune defense response including the acute phase response, complement activation, and humoral responses mediated by circulating immunoglobulins. Similar to the findings of the current study, others have shown that targeted protein biomarkers such as myeloperoxidase (MPO) and various acute phase proteins including serum amyloid A (SAA), complement factors, and alpha-1-acid glycoproteins are elevated after ultramarathon events or prolonged and intensive exercise training periods [9,18,25,32,33,34,35,36,37,38]. 

The acute phase response is a systemic reaction to environmental insults including severe stress, infection, trauma, and late-stage cancer, and involves the hepatic production of many proteins including SAA, C-reactive protein, complement proteins, antiproteases, transport proteins, and those involved with the coagulation and fibrinolytic system [39,40]. During the acute phase response, plasma levels of SAA rise to very high levels, are produced by hepatocytes and tissue macrophages, and trigger multiple signaling pathways related to phagocyte migration and inflammation [39]. Similar to the findings of the current study, SAA was elevated 48 h following the 246-km Spartathlon race in a group of ultradistance runners [9,33]. In this study, IL-6 increased immediately after the Spartathlon race and then returned to normal in contrast to SAA that remained high during the 2-day recovery period [9,33]. IL-6, which rises to high levels following stressful exercise bouts, stimulates the production of most acute-phase proteins by hepatocytes that influence one or more stages of inflammation [40]. Animal-based studies have focused on the value of measuring SAA and other acute phase proteins in monitoring physiological responses to intensified training stress [10,11,12,13,14,15,16,17]. One study of 20 Arabian horses showed that higher compared to lower pre-race serum SAA levels were strongly linked to an inability to complete 120- and 160-km endurance events [10]. 

In the current study, MPO rose strongly on the second day of recovery from the 3-day period of intensified exercise. MPO is a lysosomal protein stored in azurophilic granules of the neutrophil and during degranulation is released into the extracellular space. MPO is a biomarker of neutrophil activation and inflammation following strenuous exercise [15,16,17] and is systemically elevated in patients with coronary artery disease [25,41]. MPO was increased for at least 19 days in 42 triathletes following an Ironman triathlon, although this study lacked a suitable control group [25]. 

## 5. Conclusions

Prior studies utilized a limited number of proteins, and most were based on acute shifts in racing dogs and horses. The chief contribution of the current study was the use of a system-wide proteomics approach to define clusters of blood proteins from DBS samples that were (1) expressed acutely post-exercise and (2) chronically during 2-day recovery from a 3-day period of intensified exercise (FOR). Of 593 proteins identified, 60 proteins increased significantly after the 2.5 h exercise bout on Day 1 and 15 after each of the three days of exercise compared to rest. Thirteen of the identified proteins did not increase acutely post-exercise, but increased on the morning of day 1 and/or day 2 of recovery. Most of these proteins (acute and chronic) signaled an exercise-induced activation of innate immune function, supporting prior research demonstrating the heavy involvement of the immune system in restoring homeostasis after intense exercise [42,43]. The next step in this line of research is to test the targeted panel of FOR-related proteins defined in this study in NFOR- and OTS-based investigations with high-level athletes. The “chronic” period included in this study after FOR (2 days of recovery) could be extended for weeks and months within the NFOR and OTS context. The ultimate goal is to refine the targeted proteomics panel so that when combined with various other tools such as the TDS and workload assessment will result in a highly predictive process that will assist the coach in individualizing training regimens to prevent NFOR and OTS in athletes. 

## Figures and Tables

**Table 1:** Characteristics of study participants (N = 10 males) (mean ± SE).

Variable	Mean ± SE
Age (years)	38.3 ± 3.4
Height (m)	1.81 ± 0.02
Weight (kg)	85.6 ± 1.3
Body fat (%)	20.9 ± 2.1
VO_2max_ (mL kg^−1^ min^−1^)	42.0 ± 1.3
Maximal heart rate (beats/min)	176 ± 3.7

**Table 2:** Proteins (N = 15) increasing significantly pre-to-post-exercise (acutely) compared to rest after each of the three 2.5 h exercise sessions. Protein sizes and chromosome locations are available at https://www.uniprot.org/.

UniProt Protein	Protein Name	Basic Function
P61626	Lysozyme C	Monocyte/macrophage bacterilytic function
P08246	Neutrophil elastase	Modifies the functions of natural killer cells, monocytes and granulocytes
P59665; P59666	Neutrophil defensin 1	Antibacterial, fungicide, antiviral activity; kills by permeabilizing membrane
P80511	Protein S100-A12	Ca, Zn, Cu binding protein; prominent role, regulation inflammation/immune
P05109	Protein S100-A8	Ca, Zn binding protein; regulate inflammation/immune; chemotaxis
P49913	Cathelicidin antimicrobial peptide	Binds to bacterial lipopolysaccharides (LPS), has antibacterial activity
P12814	Alpha-actinin-1	F-actin cross-linking protein to anchor actin to intracellular structures
P60709	Actin, cytoplasmic 1	Cell motility; granulocytes
P07737	Profilin-1	Binds to actin; granulocyte motility/chemotaxis
P02776	Platelet factor 4	Released during platelet aggregation; chemokine activity; chemotaxis
P60660	Myosin light polypeptide 6	Regulatory light chain myosin; muscle development
Q96QV6; Q93077	Histone H2A types	Component of nucleosome; transcription regulation, DNA repair
P62805	Histone H4	Component of nucleosome; transcription regulation, DNA repair
P05204	Non-histone chromosomal protein HMG-17	Binds nucleosomal DNA
O95810	Serum deprivation-response protein	Targets protein kinase C-alpha on lipid rafts

**Table 3:** Proteins increasing on day 1 and/or day 2 of recovery from the 3-day exercise period compared to rest, but not acutely immediately post-exercise. Protein sizes and chromosome locations are available at https://www.uniprot.org/.

UniProt Protein	Protein Name	Function
P35542	Serum amyloid A-4 protein	Major acute phase reactant; cell chemotaxis
P05164	Myeloperoxidase	Granulocyte microbicidal activity against wide range of pathogens; production of hypochlorous acid
P07360	Complement component C8 gamma chain	Part of membrane attack complex that plays key role in immune response; forms pores in target cells
P0C0L5	Complement C4B	Non-enzymatic component C3, C5 convertases and thus essential for complement activation
P05155	Plasma protease C1 inhibitor	Crucial role in regulation of complement activation
Q14624	Inter-alpha-trypsin inhibitor heavy chain H4	Acute-phase protein involved in trauma inflammatory response
P19652	Alpha-1-acid glycoprotein 2	Transport protein; modulates immune function during the acute-phase reaction; inflammation
P10643	Complement component C7	Part of membrane attack complex that plays key role in immune response; forms pores in target cells
P02765	Alpha-2-HS-glycoprotein	Promotes endocytosis; part of acute-phase response; phagocytosis; bone mineral influence
P01834	Immunoglobulin kappa constant	Constant region of immunoglobulin heavy chains; complement activation; defense immune response; phagocytosis recognition and engulfment
P01871; P04220	Immunoglobulin heavy constant mu	Constant region of immunoglobulin heavy chains; C region; antigen binding; immune response
P08185	Corticosteroid-binding globulin	Major transport protein for glucocorticoids and progestins
P35754	Glutaredoxin-1	Glutathione activity; cell redox homeostasis

## Supplementary Materials

The following are available online http://www.mdpi.com/2227-7382/6/3/33/s1. Table S1 provides intensity data for the 593 proteins identified for each of the 16 blood samples acquired during the exercise and rest periods (XLSX).

## Abbreviations

The following abbreviations are used in this manuscript:

CV	Coefficient of variation
DIA	Data independent acquisition
DBS	Dried blood spot
FDR	False discovery rate
FOR	Functional overreaching
GEE	Generalized estimating equation
GLMM	Generalized linear mixed models
NFOR	Nonfunctional overreaching
OTS	Overtraining syndrome
RPE	Rating of perceived exertion
SAA	Serum amyloid A
STRING	Search tool for the retrieval of interacting genes/proteins
TDS	Training distress scale
VO_2_max	Maximal volume of oxygen consumption
